# Advances in molecular regulation and function of LDLR family in viral infection

**DOI:** 10.3389/fmicb.2025.1702637

**Published:** 2025-10-31

**Authors:** Qing Yao, Jun Gong, Helin Lu, Wu Liu, Liqiong Ding

**Affiliations:** ^1^School of Pharmacy, Hubei University of Science and Technology, Xianning, China; ^2^Hubei Key Laboratory of Diabetes and Angiopathy, Hubei University of Science and Technology, Xianning, China

**Keywords:** LDLR, virus infection, receptor, lipid, PCSK9

## Abstract

The low-density lipoprotein receptor (LDLR) family represents a crucial interface between cellular cholesterol homeostasis and viral pathogenesis. This review systematically examines the dual roles of these receptors in viral infections, encompassing both their well-established function as entry receptors for various viruses and their emerging role as regulators of viral replication through lipid metabolic pathways. The LDLR family mediates exogenous cholesterol uptake that supports viral proliferation while simultaneously suppressing endogenous cholesterol synthesis. This suppression triggers endoplasmic reticulum cholesterol depletion, which activates the STING-TBK1 signaling axis, thereby establishing a potent antiviral state. These opposing mechanisms reveal the complex involvement of the LDLR family in viral infections. This article aims to synthesize current understanding of these processes and explore the translational potential of targeting the LDLR-lipid-virus axis for developing novel antiviral strategies, while acknowledging the challenges in selectively modulating these dual functions for therapeutic purposes.

## 1 Overview of the LDLR family

The low-density lipoprotein receptor family consists of 14 structurally related members, including low-density lipoprotein receptor (LDLR), very low-density lipoprotein receptor (VLDLR), and LDLR-related protein (LRP), etc. These receptors typically contain extracellular ligand-binding domains (LBDs), epidermal growth factor (EGF)-like repeats, transmembrane regions, and cytoplasmic tails (Campion et al., 2020; Zhao et al., 2025; Liu et al., 2024). This conserved structure supports their central role in the specific recognition of extracellular ligands and the precise regulation of cellular lipid metabolism (Schmidt et al., 2025; Bolanle et al., 2025; Corsini et al., 2025). processes critically implicated in the pathogenesis of various diseases such as atherosclerosis and Alzheimer’s disease (Chandrashekar et al., 2024; Huang et al., 2025).

LDLR is a widely expressed transmembrane glycoprotein located on the cell surface that mediates the endocytosis of LDL particles (Sun et al., 2025). Sterol regulatory element-binding proteins (SREBPs) tightly control LDLR expression at the transcriptional level, while subtilin/kexin type 9 protein-converting enzyme (PCSK9) strictly controls LDLR expression at the post-transcriptional level to preserve cholesterol homeostasis. Low-cholesterol environments activate SREBPs, which attach to the LDLR promoter to enhance transcription (Bai et al., 2024). On the cell surface, the released PCSK9 attaches to LDLR and the LDLR-PCSK9 complex is internalized through endocytosis mediated by clathrin, which is then taken to lysosomes for degradation (Guan et al., 2025). Emerging evidence have shown that LDLR family is utilized by diverse viruses at different stages of their life cycle. As a receptor protein, certain LDLR members facilitate viral adhesion and cellular invasion, offering critical insights into the mechanisms of virus-host interactions (Monteil et al., 2024; Wani et al., 2025; Fan et al., 2025; Bhaskar et al., 2025). In addition, as core regulators of lipid metabolism, the LDLR family proteins manipulate the uptake of low-density lipoprotein cholesterol and the synthesis of endogenous cholesterol, affecting viral replication (Muzammil et al., 2024).

**Figure 1:**
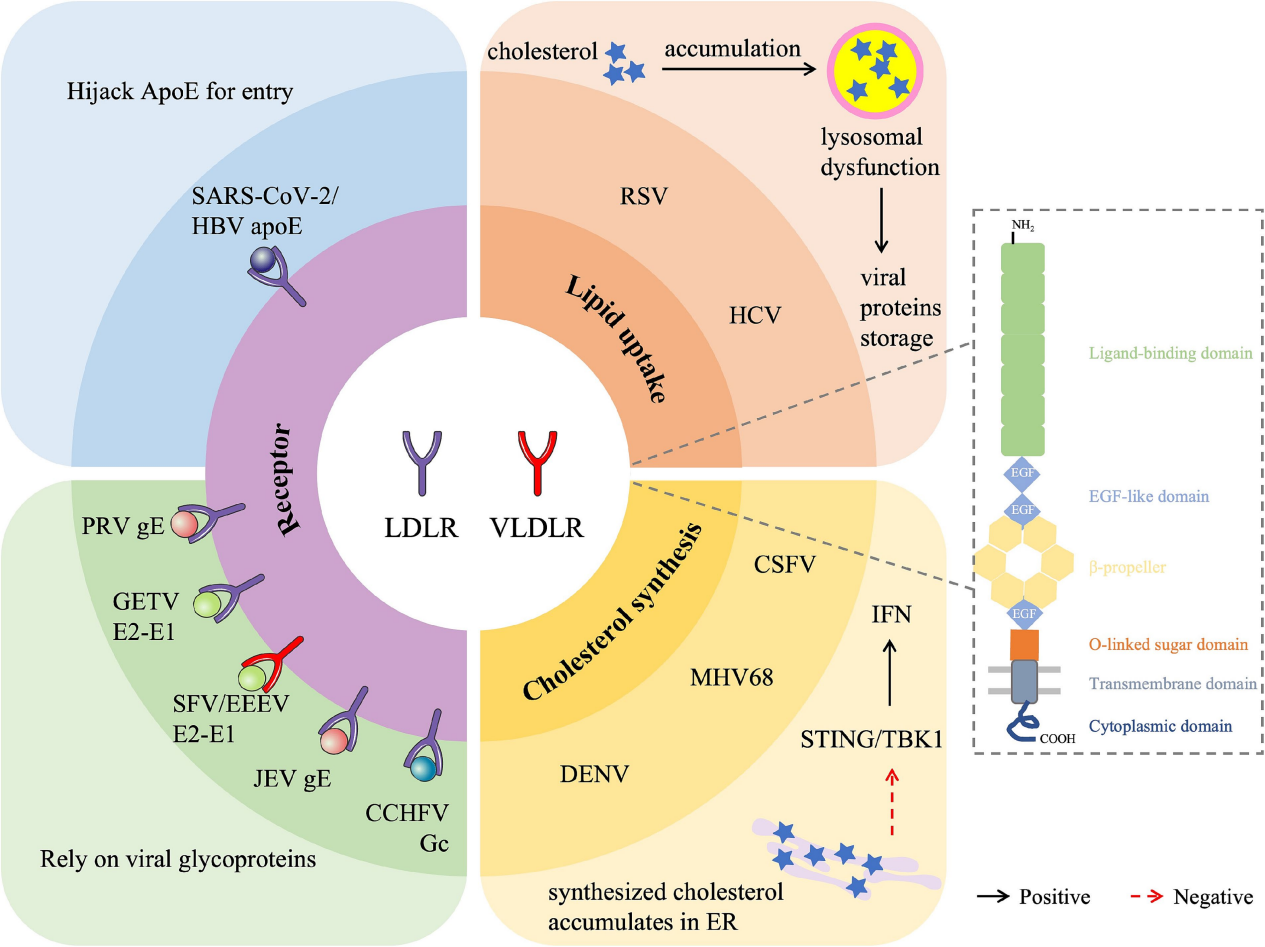
Overview of the role of LDLR family protein in viral infection. LDLR family members are involved in the regulation of various viral infections by acting as a receptor for viral entry (LDLR for SARS-CoV-2, HBV, PRV, GETV, JEV and CCHFV, VLDLR for SFV and EEEV), promoting lipid uptake (RSV and HCV) or inhibiting cholesterol synthesis (DENV, MHV68, and CSF). SARS-CoV-2, Severe acute respiratory syndrome coronavirus 2; HBV, Hepatitis B virus; PRV, Pseudorabies virus; GETV, Getah virus; SFV, Semliki Forest virus; EEEV, Eastern equine encephalitis virus; JEV, Japanese encephalitis virus; CCHFV, Crimean-Congo hemorrhagic fever virus; RSV, Respiratory syncytial virus; HCV, Hepatitis C virus; DENV, Dengue virus; CSFV, Classical swine fever virus; MHV68, Murine herpesvirus 68.

In this review, we first systematically examine the dual role of the LDLR family in viral infection, focusing on its function as a key mediator of viral entry and as a critical regulator of lipid metabolism that participates in viral replication (Figure 1). We then discuss the emerging potential of targeting LDLR family members for the development of novel antiviral therapies.

## 2 The role of LDLR family in viral infection

### 2.1 The LDLR family serves as the receptor for viral entry

The LDLR family functions as a critical entry portal for a diverse spectrum of viruses. Research has identified its involvement in the infection of human, animal, and zoonotic viruses, revealing both common strategies and distinct molecular interactions.

#### 2.1.1 Human viruses

##### 2.1.1.1 Severe acute respiratory syndrome coronavirus 2

Since the emergence of severe acute respiratory syndrome coronavirus 2 (SARS-CoV-2) in 2019, it has rapidly spread to most countries in the world, posing a huge threat to public health (Zhou et al., 2020). Starting with the RNA sequencing of the new coronavirus SARS-CoV-2, scientific research on SARS-CoV-2 has progressed rapidly, and angiotensin-converting enzyme 2 (ACE2) has been identified as receptor for SARS-CoV-2 spike protein (Hoffmann et al., 2020), but more and more information indicates that SARS-CoV-2 could still infect a variety of tissues and cells with reduced expression of ACE2 (Partridge et al., 2021; Shen et al., 2022). Therefore, there may be alternative entry routes for SARS-CoV-2. Uppal et al. discovered that both LDLR-specific siRNA and anti-LDLR antibody prevent SARS-CoV-2 infection (Uppal et al., 2023). Cui et al. demonstrated that apolipoprotein E (ApoE) mediates the entry of SARS-CoV-2 via binding to LDLR. ApoE neutralizing antibodies or knockout of LDLR can both effectively block SARS-CoV-2 infection (Cui et al., 2023). These results suggested that SARS-CoV-2 infection is dependent on LDLR.

##### 2.1.1.2 Hepatitis B virus

Hepatitis B virus (HBV) is a DNA virus that is closely related to a range of liver diseases. Although there are effective vaccines and antiviral therapies that inhibit viral replication, it is still difficult to completely cure (Xu et al., 2025). Understanding HBV infection and replication mechanisms is critical for developing new antiviral drugs. Li et al. determined the function of LDLR in HBV infection and found that LDLR monoclonal antibody can effectively inhibit HBV infection (Li and Luo, 2021). LDLR binds ApoE (Tripathi et al., 2025), and evidence shows that ApoE enrichment on the HBV envelope enhances infection (Qiao and Luo, 2019). Further studies found that LDLR effectively blocks the binding of ApoE to heparin, suggesting that LDLR may act as an HBV cell attachment receptor to bind to HBV-related ApoE and mediate HBV entry into cells.

The reliance of both SARS-CoV-2 and HBV on ApoE to engage LDLR represents a convergent evolutionary strategy, where the virus exploits a natural ligand of the receptor as a bridge for entry, rather than evolving a high-affinity viral glycoprotein for direct binding.

#### 2.1.2 Animal viruses

##### 2.1.2.1 Pseudorabies virus

Pseudorabies virus (PRV) is a swine herpesvirus that causes significant economic losses to the global swine industry (Xu L. et al., 2024). During the process of PRV attachment, LDLR co-localizes with PRV glycoprotein E (gE) to facilitate viral entry (Ma Y. X. et al., 2024). PRV upregulates LDLR expression through SREBP2, and knockdown of SREBP2 significantly reduces both LDLR and PRV gE expression. Genetic silencing of LDLR markedly attenuates PRV infection, whereas pharmacological inhibition of PCSK9, a mediator of LDLR degradation, increases viral titers. These findings demonstrate the crucial role of LDLR in PRV infection and its regulation through SREBP2-mediated transcriptional activation and PCSK9-dependent post-translational modification.

#### 2.1.3 Alphaviruses

Alphaviruses are mosquito-borne RNA viruses that are pathogenic to humans and livestock, posing a serious public health burden (Kim and Diamond, 2023). While several receptors of alphaviruses have been identified (Zhang et al., 2018; Ma et al., 2020), they do not account for the wide host range and tissue tropism of some alphaviruses, suggesting the presence of other receptors. Recent studies indicate that LDLR family proteins mediate the entry of multiple alphaviruses, including Getah virus (GETV), Semliki Forest virus (SFV), and Eastern equine encephalitis virus (EEEV) (Zhai et al., 2024; Ma et al., 2025).

##### 2.1.3.1 GETV

The ectopic expression of LDLR promotes the interaction of the E2 and E1 glycoproteins (E2-E1) of GETV with the LBD of LDLR, which in turn mediates the cellular binding and internalization of GETV (Zhai et al., 2024). Anti-LBD antibodies have been found to block GETV infection. In addition, the key amino acids that play a crucial role in virus entry in LDLR-LBD have also been identified. Specific mutations in the CR4 and CR5 domains of LDLR-LBD significantly reduce the speed at which viruses enter cells. These findings suggest that LDLR, as a cell entry receptor, increases GETV infection by promoting cell entry.

##### 2.1.3.2 SFV

The E2-E1 of SFV could also interact with the LBD of VLDLR. The ectopic expression of VLDLR promotes cell attachment and internalization, and SFV infection can be prevented by VLDLR LBD-Fc fusion protein or ligand-binding antagonist, a receptor-associated protein that binds to LDLR-associated receptors in the endoplasmic reticulum (ER) and blocks ligand binding (Clark et al., 2021). Recently, Cao et al. used cryo-electron microscopy to study the structure of SFV and VLDLR complexes, and found that VLDLR binds to multiple E1-DIII sites of SFV through its distal LDLR class A (LA) repeats, among which LA3 has the best binding affinity with SFV (Cao et al., 2023). All the above showed that VLDLR acts as an entry receptor for SFV by binding to multiple E1-DIII sites of SFV.

##### 2.1.3.3 EEEV

VLDLR has also been identified as the receptor of EEEV (Alcorn et al., 2025). Ma et al. found that the expression of LDLR promoted the binding and infection of EEEV to cells (Ma H. et al., 2024). Adams et al. resolved cryo-electron microscopy structures of the EEEV-VLDLR complex and found that EEEV uses multiple sites to bind to LA domains of VLDLR, which provides a research basis for the design of the smallest VLDLR decoy receptor (Adams et al., 2024; Yang P. et al., 2024). Recent studies have found that the W132G mutation in VLDLR disrupts LA3 binding and significantly enhances the attachment of EEEV to cells, implying that people with similar VLDLR mutations may be highly susceptible to EEEV infection (Cao et al., 2024).

In contrast to the ApoE-bridging mechanism, alphaviruses like GETV, SFV, and EEEV employ their own viral glycoproteins to directly engage the ligand-binding domains of LDLR or VLDLR. Furthermore, the preference for different family members and the precise binding interfaces, such as GETV’s use of LDLR versus SFV/EEEV’s use of VLDLR, illustrate how subtle differences in receptor usage can fine-tune viral tropism and entry efficiency.

#### 2.1.4 Zoonotic viruses

##### 2.1.4.1 Japanese encephalitis virus

Japanese encephalitis virus (JEV) is a zoonotic virus transmitted by mosquitoes, with more than 68,000 cases of Japanese encephalitis caused by JEV infection reported annually and a mortality rate of up to 30% (Yang K. et al., 2024). Studies have shown that LDLR is necessary for JEV to enter the host cells (Huang et al., 2021). Pre-incubation with LDL, the ligand of LDLR, significantly inhibited JEV internalization, and knockout of LDLR reduced JEV infection in A549 cells. In addition, co-immunoprecipitation showed that LDLR interacted with JEV envelope gE. These findings suggested that LDLR is necessary for JEV entry into host cells.

##### 2.1.4.2 Crimean-Congo hemorrhagic fever virus

Crimean-Congo hemorrhagic fever virus (CCHFV) has a wide range of hosts and can infect humans and various wild animals (Hawman and Feldmann, 2023). Human infection with CCHFV can cause severe viremia and hemorrhagic fever, with a case fatality rate as high as 40% (Oraby and Marchant, 2024). It was found that LDLR is a key receptor for CCHFV cell entry, and the interaction between CCHFV and LDLR is highly specific, and LDLR can pre-bind the surface glycoprotein Gc of CCHFV (Xu Z. S. et al., 2024). At the same time, LDLR deficiency can lead to a delay in CCHFV-induced disease (Ritter et al., 2024). This discovery has far-reaching implications for the treatment of CCHFV.

Viruses like JEV and CCHFV further exemplify the direct viral glycoprotein binding strategy, underscoring its prevalence. The collective evidence demonstrates that the LDLR family serves as a versatile portal for viral entry. The key distinction lies in the molecular strategy for receptor engagement, which can be broadly categorized into indirect mechanisms exploiting host ligands and direct mechanisms relying on viral glycoproteins. This mechanistic understanding provides a deeper rationale for considering the LDLR family as a target for broad-spectrum antiviral strategies.

### 2.2 The role of the LDLR family in viral infection by regulating lipid metabolism

#### 2.2.1 The LDLR family promotes viral infection by mediating lipid uptake

LDLR is responsible for taking up cholesterol-rich lipoproteins from the blood (Ben-Naim et al., 2022), and the proliferation of some viruses is highly dependent on host exogenous lipid uptake (Albano et al., 2025). Therefore, LDLR can also be involved in viral infections by regulating lipid uptake.

##### 2.2.1.1 Respiratory syncytial virus

**Figure 2:**
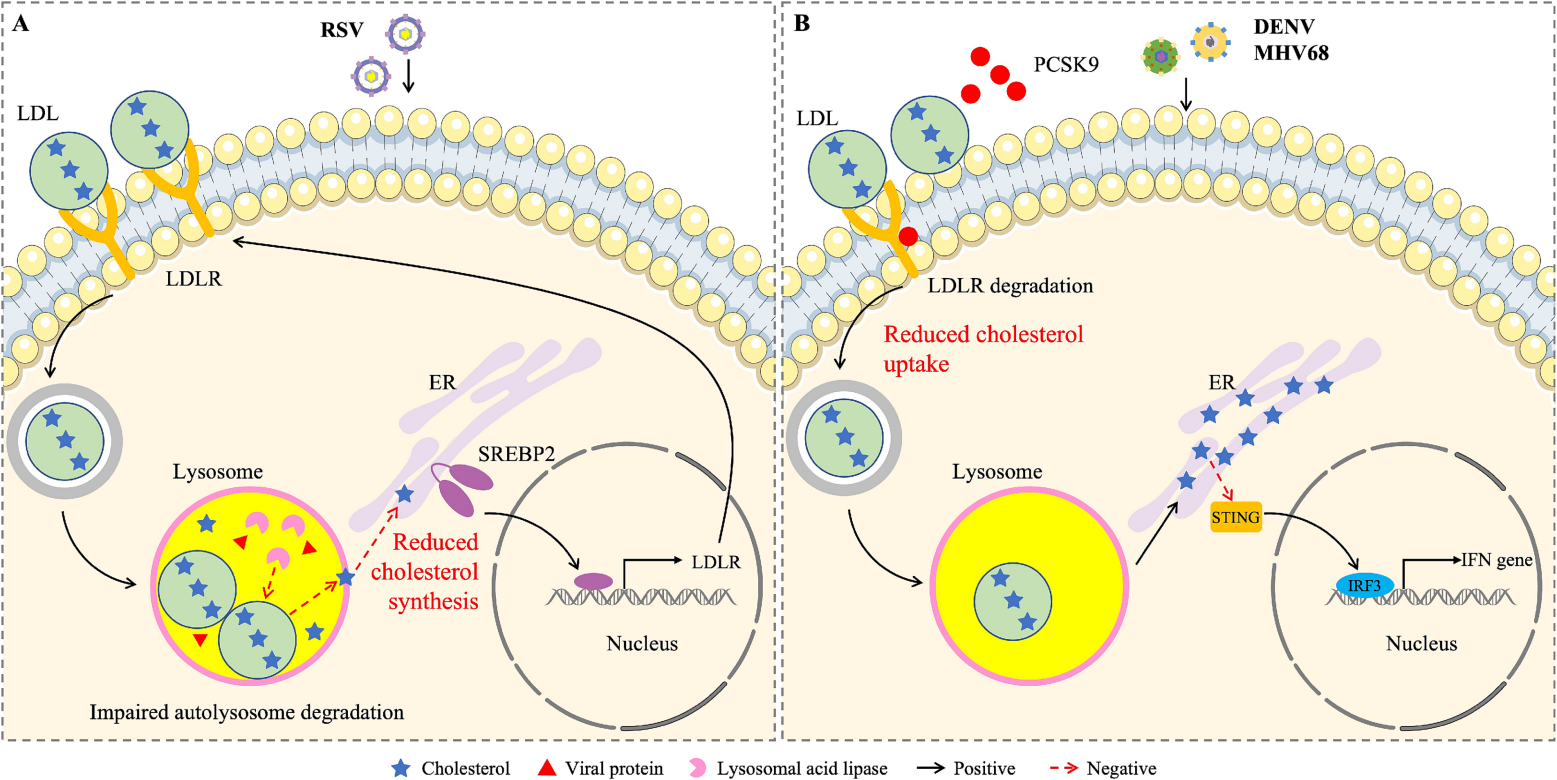
Schematic diagram of the effects of cholesterol from different sources on viral infections. **(A)** RSV blocks the transport of cholesterol from lysosome to ER by reducing the activity of lysosomal acid lipase, activates the SREBP2-LDLR axis, promotes the uptake and accumulation of exogenous cholesterol in the lysosome of infected cells, leads to lysosomal dysfunction, which favors the storage of viral proteins. **(B)** DENV and MHV68 infections reduce the circulation of LDLR and cholesterol uptake by inducing the expression of PCSK9. The newly synthesized cholesterol accumulates in ER and inhibits the activation of STING and type I IFN.

Respiratory syncytial virus (RSV) is the most common cause of respiratory diseases such as bronchitis and pneumonia (Ponce et al., 2025). Despite many efforts in the development of anti-RSV drugs, there are currently no therapeutic intervention measures (Paes et al., 2020). Studies have shown that cholesterol is necessary for the infectivity and stability of RSV (Pastey et al., 2025). On this basis, Chen et al. investigated the role of LDLR in RSV infection and found that RSV infection activates the SREBP2-LDLR axis, promoting lipid uptake and the accumulation of exogenous cholesterol in lysosomes (Chen et al., 2024). At the same time, RSV inhibited the transport of cholesterol from lysosome to ER by down-regulating lysosomal acid lipase activity, thus blocking autophagy flux and promoting viral replication (Figure 2A). Knocking out LDLR effectively inhibits RSV infection *in vivo*. These results indicate that RSV infection promotes lipid uptake by activating LDLR, thereby facilitating RSV replication. LDLR may be a potential target for anti-RSV drugs.

##### 2.2.1.2 Hepatitis C virus

Hepatitis C virus (HCV) is a single-stranded RNA virus that infects 2–3% of the global population and has become a major health crisis (Zhang M. et al., 2024). It is reported that LDLR is associated with HCV susceptibility and immune evasion (Steba et al., 2019; Olesen et al., 2025; Real et al., 2019). The uptake of cholesterol-rich LDL particles by LDLR plays a significant role in HCV replication, as lipids play a key role in HCV infection (Muzammil et al., 2024; Caron et al., 2019). HCV has been reported to stimulate the expression of LDLR, leading to an increase in the uptake of lipoprotein particles and thereby facilitating viral propagation (Syed et al., 2014). HCV upregulates the transcription of the LDLR gene through SREBPs, and simultaneously degrades PCSK9 through the proteasome pathway, inhibiting its degradation effect on the LDLR protein. Ectopic expression of wild-type PCSK9 has a negative impact on HCV replication. These results indicate that HCV promotes lipid uptake and viral proliferation by regulating the expression of LDLR.

In addition, some studies have also shown that LDLR redundantly involved in the entry of HCV (Yamamoto et al., 2016; Zapatero-Belinchón et al., 2021). Compared with the sole deficiency of scavenger receptor class B type 1 (SR-B1) or LDLR, the deficiency of SR-B1 and LDLR causes greater damage to HCV entry. The exogenous expression of SR-B1 and LDLR restored the entry of HCV in SR-B1 and LDLR double knockout cells. Therefore, LDLR is involved in HCV infection at multiple stages of the viral life cycle, including the entry and replication of the virus.

#### 2.2.2 The LDLR family combat viral infection by inhibiting endogenous cholesterol synthesis

Cellular cholesterol homeostasis is maintained through a balance between exogenous uptake mediated by receptors such as LDLR and endogenous biosynthesis controlled by the SREBP2 pathway. An increase in LDLR-mediated cholesterol influx suppresses SREBP2 activation, thereby downregulating the expression of cholesterol synthetic enzymes and reducing *de novo* cholesterol production (Camps et al., 2025). This deliberate limitation of endogenous synthesis results in a specific reduction of cholesterol within the ER. The depleted ER cholesterol pool acts as a critical signal that directly promotes the activation of the STING-TBK1 signaling axis (Zhang B. C. et al., 2024). Activated TBK1 phosphorylates the transcription factor IRF3, which dimerizes and translocates to the nucleus to drive the expression of type I interferons (Scoles and Pulst, 2024). The subsequent induction of a broad spectrum of interferon-stimulated genes establishes a robust intracellular antiviral state, illustrating a key mechanism by which the LDLR family exerts its antiviral function.

#### 2.2.3 Human viruses

##### 2.2.3.1 Dengue virus

Dengue virus (DENV) is transmitted by Aedes mosquitoes, and infected individuals exhibit a range of clinical symptoms, ranging from febrile dengue to severe dengue, which can lead to up to 20% mortality (Tarazona-Castro et al., 2025). Studies have shown that DENV could down-regulate LDLR protein levels by increasing PCSK9 expression and reduce cholesterol uptake, thereby enhancing DENV infection (Gan et al., 2020). LDLR inhibited cholesterol resynthesis by mediating cholesterol uptake, thereby preventing the damaging effect of ER cholesterol levels on STING-mediated IFN induction (Figure 2B). The infection of DENV is also enhanced by down-regulating the expression of LRP1 (Tree et al., 2019). LRP1 decreased intracellular cholesterol content and inhibited DENV replication. Therefore, these results indicate that DENV reduces the expression of LDLR and LRP1 to promote infection.

##### 2.2.3.2 Human cytomegalovirus

Human cytomegalovirus (HCMV) is a beta herpesvirus that infects more than 90% of total population, leading to the establishment of a lifetime viral latency (Ma et al., 2023). LRP1 expression increases during HCMV infection, reducing viral cholesterol and infectivity (Gudleski-O'Regan et al., 2012). The increased expression of LRP1 may be a defensive response to HCMV infection, suggesting that LRP1 limits the infectivity of HCMV.

#### 2.2.4 Animal viruses

##### 2.2.4.1 Murine herpesvirus 68

Gamma herpesviruses are a family of DNA viruses that pose a significant burden to human health (Singh et al., 2024). Murine herpesvirus 68 (MHV68) is a natural rodent pathogen that provides an easy-to-handle experimental system to define gamma herpesvirus-host interactions (Riggs et al., 2021). LDLR has been reported to counteract MHV68 replication by inhibiting endogenous cholesterol synthesis (Aurubin et al., 2021). LDLR protein level was reduced by MHV68 to resist the antiviral effect of LDLR. LDLR knockout in macrophages, but not mouse embryonic fibroblasts, caused an increase in endogenous cholesterol synthesis, leading to viral gene expression. In conclusion, LDLR inhibits the replication of MHV68.

##### 2.2.4.2 Classical swine fever virus

Classical swine fever virus (CSFV) is one of the main pathogens of pigs, and infection with highly virulent strains can lead to high mortality (Gao et al., 2025). Zou et al. found that CSFV infection induces reprogramming of cholesterol metabolism in host cells, thereby promoting replication (Zou et al., 2022). Mechanically, CSFV disrupts the type I IFN response by up-regulating the expression of PCSK9, blocking the uptake of exogenous cholesterol by LDLR, and enhancing the synthesis of endogenous cholesterol. These results indicate that LDLR inhibits cholesterol biosynthesis and impairs the replication of CSFV. However, this study did not provide evidence to prove that the depletion of LDLR has a direct impact on the replication of CSFV. Recent studies have demonstrated that LDLR-specific antibodies significantly inhibit CSFV infection, while the viral titer in cells with LDLR overexpression increased significantly (Leveringhaus et al., 2024). This study reveals that the replication of CSFV depends on LDLR, which is inconsistent with the above results. This difference may be related to the different cell lines used, virus strains, and infection times. The former uses the PK15 cell line and Shimen strain, and the viral titer is measured 48 h after infection, while the latter uses the SPEV cell line and Kozlov strain, and the titer is evaluated 20 h after infection. However, the synthesis of cholesterol may take more time. The exact role of LDLR in CSFV infection still needs to be further clarified. Current data suggest that it may be virus strain or cell environment dependent.

It is worth noting that viruses exhibit significant differences in their reliance on host cholesterol metabolism during replication, which may be partly attributable to variations in the duration of their replication cycles and fundamental distinctions in the utilization of cholesterol from different sources (GBD 2021 HIV Collaborators, 2024; Farías et al., 2024). RNA viruses (such as RSV and HCV) usually have short replication cycles, so they prefer to use LDLR-mediated exogenous cholesterol uptake pathways to quickly obtain lipid resources. Studies have shown that RSV promotes the uptake and accumulation of exogenous cholesterol in lysosomes, inhibits autophagosome degradation, and thus promotes the accumulation of RSV fusion proteins (Chen et al., 2024). Similarly, HCV relies on LDLR-mediated uptake of lipoprotein particles to promote viral propagation (Syed et al., 2014). In contrast, DNA virus such as MHV68 has longer replication cycles and require a continuous and stable supply of cholesterol to support the construction of large-scale viral factories and the assembly of progeny virions. MHV68 infection relies on the host’s newly synthesized cholesterol to supports virus replication (Aurubin et al., 2021). It is worth noting that the infection of some RNA viruses (e.g., DENV) relies on the subcellular localization of cholesterol rather than the total cellular cholesterol level. Cholesterol taken up by LDLR is distributed in various cellular compartments, while resynthesized cholesterol enriches in the ER, which inhibits the activation of IFN. Therefore, the infection of DENV is also associated with the synthesis of endogenous cholesterol (Gan et al., 2020; Zou et al., 2022). Still, elucidating the precise mechanisms behind this duality remains a critical area for future research.

## 3 Therapeutic potential and challenges

LDLR family plays a critical role in viral infections, making it a promising target for novel antiviral therapies. Current strategies include blocking LDLR-virus interactions through antibodies, genetic modulation of LDLR expression, or small-molecule inhibitors. LDLR antibodies or LDLR knockout have been shown to inhibit the infection of diverse viruses, including human viruses [SARS-CoV-2 (Uppal et al., 2023), HBV (Li and Luo, 2021), and RSV (Chen et al., 2024)], animal viruses [PRV (Ma Y. X. et al., 2024)], and zoonotic agents [JEV (Huang et al., 2021) and CCHFV (Ritter et al., 2024)]. Transcriptional suppression of LDLR via SREBP2 knockdown also inhibits infection by viruses such as PRV (Ma Y. X. et al., 2024). Small molecules like berbamine reduce plasma membrane LDLR levels to confer JEV resistance (Huang et al., 2021), the natural compound Bruceine A blocks viral adsorption and internalization via lysosomal-mediated LDLR degradation (Zuo et al., 2025), and bovine lactoferrin competes for LDLR binding to inhibit viral entry (Chien et al., 2008), demonstrating their potential as broad-spectrum viral entry inhibitors.

However, therapeutic targeting of LDLR must account for virus- and cell- or tissue-specific outcomes. While PCSK9-mediated degradation of LDLR inhibits RSV and HCV infection (Chen et al., 2024; Syed et al., 2014), PCSK9 may conversely promote DENV and MHV68 replication by elevating endogenous cholesterol synthesis and facilitating viral gene expression (Gan et al., 2020; Aurubin et al., 2021). These divergent effects underscore the importance of virus-dependent modulation. Furthermore, cell type-specific responses add another layer of complexity, as the regulation of lipid and cholesterol metabolism is cell type-dependent. Elevated LDLR expression correlates with enhanced interferon production and antiviral activity in macrophages (Lai et al., 2025). LDLR inhibits the replication of MHV68 in primary macrophages while lacks antiviral effects in mouse embryonic fibroblasts (Aurubin et al., 2021). Importantly, considering that mouse plasma cholesterol is transported by high-density lipoprotein rather than low-density lipoprotein (Gordon et al., 2015), LDLR may not have a significant impact on the infection of these viruses in mouse models. This duality presents a challenge, as therapeutic strategies targeting LDLR could yield opposite effects (enhancing vs. inhibiting infection) depending on the virus and cell or tissue environments.

Given these complexities, the development of LDLR-targeting antiviral agents faces considerable challenges, particularly in achieving cell-specific delivery and minimizing off-target effects on lipid metabolism and immune function. Future efforts should focus on elucidating tissue-specific regulatory mechanisms and developing precision strategies that avoid unintended consequences while effectively disrupting viral life cycles.

## 4 Conclusion and future perspectives

The LDLR family plays a critical role in diverse viral infections, making it a promising therapeutic target (Table 1). As receptors for virus entry, members of LDLR family mediate the entry of various viruses, including DNA (e.g., HBV, PRV) and RNA viruses (e.g., SARS-CoV-2, GETV, SFV, EEEV, JEV, CCHFV). In addition, LDLR family members also play a key role in viral infections by participating in lipid metabolism. The disturbance of lipid metabolism homeostasis can affect the viral infection process. On the one hand, lipids play an important role in the replication of the viral genome and the assembly of viral particles (Swain et al., 2023; Sidorkiewicz, 2021). On the other hand, viruses can utilize the lipid mechanisms of the host to support their life cycles and weaken the host’s immune response (Proto et al., 2021). Lipid uptake by LDLR family leads to the distribution of cholesterol in different cell compartments, while the resynthesized cholesterol causes lipid enrichment in the ER. With the enrichment of cholesterol in the ER, the activation of STING in the ER is inhibited during viral infection.

**Table 1:** Summary of LDLR roles in the life cycle of diverse viruses.

LDLR function	Virus	Experimental evidence	References
Entry	SARS-CoV-2	ApoE mediates the entry of SARS-CoV-2 via binding to LDLR. LDLR-specific siRNA and anti-LDLR antibody prevent SARS-CoV-2 infection	Uppal et al. (2023), Cui et al. (2023)
	HBV	The binding of LDLR with ApoE of HBV mediates HBV entry into cells. LDLR antibody effectively inhibits HBV infection	Li and Luo (2021), Tripathi et al. (2025), Qiao and Luo (2019)
	PRV	LDLR co-localizes with PRV gE, promoting virus entry. Genetic silencing of LDLR attenuates PRV infection	Ma Y. X. et al. (2024)
	GETV	The ectopic expression of LDLR promotes the interaction between GETV and LDLR, mediating the cellular binding and internalization of GETV	Zhai et al. (2024)
	SFV	SFV interacts with the LBD of VLDLR, and the ectopic expression of VLDLR promotes cell adhesion and internalization	Clark et al. (2021), Cao et al. (2023)
	EEEV	EEEV binds to VLDLR with multiple sites	Adams et al. (2024), Yang P. et al. (2024), Cao et al. (2024)
	JEV	LDL pre-incubation inhibited the internalization of JEV, and LDLR knockout reduced JEV infection. LDLR interacted with gE of JEV	Huang et al. (2021)
	CCHFV	CCHFV interacts specifically with LDLR. The deficiency of LDLR can lead to the delay of diseases induced by CCHFV	Xu Z. S. et al. (2024)
Replication-exogenous lipid uptake	RSV	RSV infection activates the SREBP2-LDLR axis, promoting lipid uptake and the accumulation of exogenous cholesterol in lysosomes, and facilitating viral replication	Chen et al. (2024)
	HCV	HCV stimulates the expression of LDLR, leading to an increase in the uptake of lipoprotein particles and thereby facilitating viral propagation	Syed et al. (2014)
Replication-endogenous cholesterol synthesis	DENV	DENV infection downregulates LDLR protein levels, reducing LDLR-mediated cholesterol uptake, which induces the resynthesis of cholesterol, increasing the cholesterol levels in the ER, thereby impairing the STING-mediated IFN induction	Gan et al. (2020)
	HCMV	LRP1 limits the infectivity of HCMV by controlling the availability of cholesterol in the viral particle envelope. Inhibiting LRP1 increases intracellular cholesterol and subsequently boosts the production of infectious viruses	Gudleski-O'Regan et al. (2012)
	MHV68	LDLR-mediated cholesterol influx into cells diminishes endogenous cholesterol synthesis, induces ISG expression, thereby enhancing antiviral responses. In the absence of LDLR expression, endogenous cholesterol synthesis increases, promoting MHV68 viral gene expression	Aurubin et al. (2021)
	CSFV	CSFV infection upregulates the expression of PCSK9, blocks the uptake of exogenous cholesterol by LDLR, enhances the biosynthetic pathway of cholesterol, and thereby disrupts the IFN response of PK15 cells LDLR-specific antibodies significantly inhibit CSFV infection in SPEV cells	Zou et al. (2022), Leveringhaus et al. (2024)

Given the conserved involvement of LDLR family members in diverse viral entry and replication mechanisms, they constitute a critical node in virus-host interactions and characterizing the function of LDLR should be a key component of the initial response to emerging viral pathogen. Acquired immunodeficiency syndrome caused by human immunodeficiency virus (HIV)-1 remains a major challenge to global public health (GBD 2021 HIV Collaborators, 2024). Cholesterol is crucial for the replication of HIV because the virus enters and exits target cells through lipid rafts (Sviridov et al., 2020). Recent studies have shown that the level of circulating PCSK9 is elevated in HIV-positive individuals and inhibiting PCSK9 has been proven to prevent the infection of HIV (Zanni et al., 2017). Compared with HIV-negative controls and positive patients without lipodystrophy, the LDLR levels in monocytes of the liver and blood of HIV-positive lipodystrophy patients were downregulated (Petit et al., 2002). However, the exact role and mechanism of LDLR in HIV infection still need to be further studied.

The bidirectional regulatory effect of LDLR in viral infections has sparked extensive discussions and controversies in the academic community. From a mechanistic perspective, this difference mainly stems from the differentiated utilization strategies of different viruses on cholesterol metabolic pathways, but existing research still faces several key challenges. Firstly, there are limitations of the experimental model. Currently, the vast majority of mechanism studies rely on transformed cell lines, but the LDLR expression levels and regulatory mechanisms of these cells are significantly different from those of primary cells. Animal models also have species-specific issues. Mouse LDLR and human LDLR are not functionally homologous. Furthermore, the complexity of the metabolic network poses a significant challenge to research. When LDLR inhibits endogenous cholesterol synthesis, cells may compensate by upregulating other receptors such as SR-BI, thereby masking the true function of LDLR.

With the advancement of technologies such as cryo-electron microscopy, metabolomics and gene editing, we are expected to clarify the interaction details between LDLR and different viral proteins at the molecular level. Meanwhile, integrating multi-dimensional omics data with artificial intelligence may help us discover new regulatory nodes. The bidirectional regulation phenomenon of LDLR in viral infection vividly interprets the complexity of host-pathogen interaction and also provides a unique perspective for us to understand the coevolution of cellular metabolism and immune defense.
